# LeishCyc: a biochemical pathways database for *Leishmania major*

**DOI:** 10.1186/1752-0509-3-57

**Published:** 2009-06-05

**Authors:** Maria A Doyle, James I MacRae, David P De Souza, Eleanor C Saunders, Malcolm J McConville, Vladimir A Likić

**Affiliations:** 1 Department of Biochemistry and Molecular Biology, University of Melbourne, Parkville, VIC 3010, Australia; 2 Bio21 Molecular Science and Biotechnology Institute, University of Melbourne, Parkville, VIC 3010, Australia

## Abstract

**Background:**

*Leishmania *spp. are sandfly transmitted protozoan parasites that cause a spectrum of diseases in more than 12 million people worldwide. Much research is now focusing on how these parasites adapt to the distinct nutrient environments they encounter in the digestive tract of the sandfly vector and the phagolysosome compartment of mammalian macrophages. While data mining and annotation of the genomes of three *Leishmania *species has provided an initial inventory of predicted metabolic components and associated pathways, resources for integrating this information into metabolic networks and incorporating data from transcript, protein, and metabolite profiling studies is currently lacking. The development of a reliable, expertly curated, and widely available model of *Leishmania *metabolic networks is required to facilitate systems analysis, as well as discovery and prioritization of new drug targets for this important human pathogen.

**Description:**

The LeishCyc database was initially built from the genome sequence of *Leishmania major *(v5.2), based on the annotation published by the Wellcome Trust Sanger Institute. LeishCyc was manually curated to remove errors, correct automated predictions, and add information from the literature. The ongoing curation is based on public sources, literature searches, and our own experimental and bioinformatics studies. In a number of instances we have improved on the original genome annotation, and, in some ambiguous cases, collected relevant information from the literature in order to help clarify gene or protein annotation in the future. All genes in LeishCyc are linked to the corresponding entry in GeneDB (Wellcome Trust Sanger Institute).

**Conclusion:**

The LeishCyc database describes *Leishmania major *genes, gene products, metabolites, their relationships and biochemical organization into metabolic pathways. LeishCyc provides a systematic approach to organizing the evolving information about *Leishmania *biochemical networks and is a tool for analysis, interpretation, and visualization of *Leishmania *Omics data (transcriptomics, proteomics, metabolomics) in the context of metabolic pathways. LeishCyc is the first such database for the Trypanosomatidae family, which includes a number of other important human parasites. Flexible query/visualization capabilities are provided by the Pathway Tools software and its Web interface. The LeishCyc database is made freely available over the Internet .

## Background

Protozoan parasites comprise a highly divergent group of eukaryotes that cause a range of debilitating diseases in humans, including malaria, leishmaniasis, African sleeping sickness, and Chagas' disease. *Leishmania *spp. are sandfly transmitted protozoan parasites (family Trypanosomatidae) and are the etiological agent of leishmaniasis. Leishmaniasis refers to a spectrum of diseases ranging from self-healing cutaneous lesions to debilitating mucocutaneous and lethal visceral infections. It is estimated that more than 12 million people have active leishmaniasis, and 350 million people are at risk, making *Leishmania *the most important parasitic disease after malaria (World Health Organization). No vaccines against leishmaniasis exist; high toxicity and cost of current treatments and the emergence of drug resistant parasite strains point to the urgent need for novel drug targets.

A detailed understanding of *Leishmania *metabolism would open new avenues for the development of new drugs, and also lead to a greater understanding of how these parasites adapt to nutrient environments in the sandfly and mammalian hosts. For example, the flagellated promastigote stages of the parasite that develop in the digestive tract of the sandfly vector obtain nutrients from the sugar rich blood meal and plant saps upon which the sandfly feeds [1]. In contrast, the mammalian infective amastigote stages develop within the sugar poor environment of the phagolysosome of macrophages and some other phagocytic cells and may exploit a variety of other carbon sources [2,3]. The recent sequencing of the genomes of three *Leishmania *species (*L. major*, *L. infantum*, *L. braziliensis*) has provided the first blueprints of the metabolic potential of these parasites [4-6]. Recently, a systems approach was used to generate a metabolic network for the *L. major *Friedlin strain and make predictions about essential genes and pathway robustness [7]. However, more than 65% of the protein-encoding sequences in the *Leishmania *genome cannot yet be assigned a function based on homology searches, and therefore it is likely that *in silico *models will need to be substantially improved as new metabolic pathways are identified.

The major database for *Leishmania *genomic data is the GeneDB genome resource, established by the Sanger Institute [8-10], and soon to be accessible via the Eukaryotic Pathogens Database Resource (EuPathDB). GeneDB was initially developed to store genomic data for *T. brucei*, *L. major*, and *S. pombe*, and was later expanded to include curated data for a number of other organisms, including bacteria, fungi, and protozoa [9,10]. GeneDB allows gene finding, protein feature predictions, and searches against customized and protein domain/families databases. It provides a number of useful tools for querying genomic data, including plain text searches, BLAST searches, regular expressions enabled motif searches, and AmiGO browsing of genes [10]. Although GeneDB is an important resource for the *Leishmania *community, it does not integrate genomic data into biochemical networks [8-10]. Kyoto Encyclopedia of Genes and Genomes (KEGG) integrates genomic, chemical, and functional information for a number of organisms [11,12]. Release 48.0 of KEGG contains 91,648 reference pathways, and genomic information from 100 eukaryotes, 709 bacteria, and 52 archaea. While this top-down approach facilitates integration of all available information and easy visual inspection of pathways in different organisms, the lack of organism specialization often means that, for more obscure organisms specific information is not easily accessible, and in some cases, not included. A different approach has been taken by the BioCyc project [13], which is built around the ontology developed to describe biological functions on a cellular and molecular level [14]. In contrast to the centralized approach used by the KEGG database, the BioCyc databases are highly distributed. The BioCyc project consists of MetaCyc (a reference database of metabolic pathways [15-18]) and a set of organism-specific databases which describe genes, gene products, metabolites, their relationships and organization into metabolic pathways [13]. MetaCyc contains experimentally elucidated metabolic pathways from a variety of organisms [17,18]. A number of organism-specific BioCyc databases are under active development and curation [16,19-24].

In this work, we report on the development and use of LeishCyc, the pathway/genome database for *L. major *based on the BioCyc ontology. The initial build of LeishCyc was based on the genome sequence of *L. major *[4] and the Wellcome Trust Sanger Institute genome annotation. Subsequently, LeishCyc was curated based on literature searches and our own experimental and bioinformatics studies. This included: (*a*) annotation and assignment of additional enzymes; (*b*) checking, deletion, creation, and modification of existing reactions and pathways; and (*c*) assignment of evidence codes and literature citations.

## Construction and content

### Initial build

The *L. major *Friedlin genome is 32.8 Mb in size, with a karyotype of 36 chromosomes. The genome data (version 5.2) was downloaded from the Sanger Institute public database . The Pathway Tools component of PathoLogic was used to build the initial version of LeishCyc from the genomic data [25]. PathoLogic requires the sequence for each genomic element (a chromosome in this case), and associated annotation file. The chromosome sequences were extracted from 36 corresponding Sanger XML files, and were edited and reformatted with in-house developed programs. The *L. major *genome annotation originally provided by Sanger was in the Artemis format, and we used Artemis to convert the annotation file for each chromosome into the GenBank format. The resulting GenBank files were edited to add headers and subsequently the automated trial and build procedure was performed in PathoLogic.

The *L. major *genome contains 8283 genes predicted to encode polypeptides. The Pathway Tools software matched 574 of these genes to MetaCyc reactions based on the presence of an EC number in the gene annotation. Another 267 polypeptides, where no EC number was supplied, were matched to reactions based on their annotated name. The automated build resulted in 841 enzymes predicted for *L. major*. After the initial build, the list of 'probable enzymes' was constructed. Probable enzymes were gene products predicted to be enzymes but which could not be matched to any particular reaction by PathoLogic. These entries were manually reviewed and assigned to reactions where possible. There were 328 probable enzymes predicted for *L. major *and 148 were assigned to reactions by manual review.

### Refinement and curation

Extensive manual curation of the database was performed based on literature search, and in-house experimental and bioinformatics studies. This included verification of enzymes and reactions deduced from the original genome annotation, refinements and improvement in the annotation of genes, enzymes, reactions and pathways, assignment of evidence codes, and inclusion of literature citations. At present LeishCyc contains 1027 enzymes and 566 metabolites organized into 704 enzymatic reactions, 37 transport reactions, and 143 metabolic pathways (Table 1).

**Table 1:** Summary of LeishCyc statistics after the initial automated build, and current status after manual curation.

**Category**	**Initial build**	**Current status**
Genome size (bp)	32,816,678	32,816,678
Polypeptides	8284	8286
tRNAs	83	83
Pathways	215	143
Enzymatic Reactions	1062	704
Transport Reactions	6	37
Enzymes	841	1027
Transporters	23	47
Compounds	795	566
Protein Complexes	2	6
Citations	117	317

Only pathways present in MetaCyc can be automatically incorporated into the pathway database by Pathway Tools [25]. As MetaCyc contains predominantly bacterial and plant pathways, some pathways known to be present in *Leishmania *spp. were not present in the initial LeishCyc build. For example, MetaCyc lacked pathways involved in the assembly of the major surface glycoconjugates of *Leishmania*, including the biosynthesis of glycosylphosphatidylinositols (GPIs) and related glycolipids, and the assembly of complex phosphoglycans on the cell surface and secreted proteins and glycolipids [26]. These and other new pathways were therefore manually created for LeishCyc based on the literature references. In addition, it was necessary to modify some of the automatically imported pathways in order to accurately represent known metabolic pathways in *Leishmania *spp. For example, the pathways for dolichyl-diphosphooligosaccharide and fatty acid biosynthesis were modified to reflect what has been experimentally observed or predicted for *Leishmania *[27,28]. In total, 66 pathways were created or modified in LeishCyc [see Additional file 1]. We have also added links between the LeishCyc pathways to show how they connect to each other.

After the initial build of LeishCyc, it was necessary to review the pathways and remove false-positive predictions [19]. All pathways were reviewed, and those deemed to be supported by weak evidence were removed. For example, a pathway was removed if it did not contain any enzymes that were unique to the pathway and there was no experimental evidence for the pathway existence in the *Leishmania *spp. In some cases, pathways were deleted and replaced with *Leishmania*-specific pathways. For example, two pathways for phospholipid biosynthesis were present after the initial build (phospholipid biosyntheses I and II). These were replaced with *Leishmania*-specific pathways for phospholipid biosynthesis (ester phospholipid biosynthesis and ether phospholipid biosynthesis) [29,30]. In total, 128 pathways were removed from LeishCyc after the initial build [see Additional file 2].

Our own experimental work was used to add and verify some of the information present in LeishCyc. For example, GC-MS analysis of polar metabolites from cultured *L. major *promastigotes revealed the presence of several metabolites (i.e. glucitol and glycerol 2-phosphate) for which exogenous sources or biosynthetic enzymes were lacking, indicating the presence of new or unanticipated reactions. Recent analyses of sugar phosphates using high resolution Fourier-transform ion cyclotron resonance (FT-ICR) mass spectrometry, identified a novel mannose cyclic phosphate that is the primer for the major intracellular reserve carbohydrate of *Leishmania*, linear polymers of mannose which we have now termed mannogen [31,32]. While none of the enzymes involved in the assembly of the mannogen primer or downstream steps have been identified, the biochemically delineated steps have been incorporated into LeishCyc.

Targeted bioinformatics studies were used to aid curation and improve the LeishCyc annotations. For example, the original genomic annotation implied only two enzymes to participate in the pathway 'dolichyl-diphosphooligosaccharide biosynthesis'. Literature review has shown that this pathway is indeed present in *Leishmania *spp. [27], and hence our bioinformatics studies were directed towards identifying genes coding for missing enzymes of this pathway. We used hidden Markov models (HMMs) to identify the *L. major *genes encoding each of the mannosyltransferases in this pathway (ALG1, ALG2, ALG3, ALG9, and ALG11). Sequences that had been characterised in other organisms were used to build HMMs for each gene product. These models were then used to scan predicted *L. major *proteins to identify the most likely candidate for each individual ALG gene. The functional assignments made based on bioinformatics studies were documented in the annotations with the appropriate evidence codes (see below).

### Use of literature to annotate LeishCyc genes and proteins

The functions of a number of *Leishmania *spp. genes have been identified in the literature since the *L. major *genome was published. As a result, these genes were not accurately annotated in the LeishCyc automated build which relied on the original annotation of the *L. major *genome project. We used extensive searches and manual reviews of published literature to incorporate additional *Leishmania *genes, proteins, enzymes, and transporters in LeishCyc. If a gene had been identified in *L. major*, the published accession number was used to identify the gene in LeishCyc. In some instances, we have judged the quality of the published information and entered the information accordingly. For example, 25 new annotation refinements were proposed for the *L. major *genome based on weak similarity using BLAST searches [7]. One of these genes (LmjF31.1780) was identified as 'sphingosine *N*-acyltransferase' based on the similarity to *Cryptococcus neoformans *sphingosine *N*-acyltransferase (E-value of 4 × 10^-6^) although a protein BLAST search of the NCBI non-redundant database returns a list with over a hundred hits with similar or better E-value, including *Trypanosoma brucei *and *Trypanosoma cruzi *proteins of unknown function (E-value of ~4 × 10^-70^). In such cases, where we believed that further evidence is needed to firmly support the proposed annotation, we have quoted the literature source and the proposed annotation, while retaining the original 'unknown' function associated with the database entry. In cases where it was deemed that computational evidence was sufficiently strong, the new functional annotation was introduced with the appropriate evidence code, as described below.

In addition to identifying published *L. major *genes, we also identified *L. major *orthologs of enzymes and transporters that have been characterized in other *Leishmania *species and trypanosomatid species such as *T. brucei *and *T. cruzi*. The *L. major *orthologs were identified by a systematic Needleman-Wunsch alignment of the given sequence against *L. major *predicted proteins with a processing pipeline built in-house. The results of Needleman-Wunsch alignment were manually reviewed for highest similarities and, if deemed appropriate, the *L. major *gene encoding the protein was annotated as the predicted ortholog (see below for the explanations of the evidence code assignments). In such cases, the percent sequence identity and/or similarity that the *L. major *sequence shared with the known homologous sequence was recorded in the LeishCyc annotation entry. For example, the *myo*-inositol transporter (MIT) has been characterized in *L. donovani*, but had not been identified *L. major*. The Needleman-Wunsch alignment of the *L. donovani *sequence against all *L. major *peptides identified LmjF24.0680 as the clear candidate for this protein in *L. major*, and the peptide with the greatest similarity to *L. donovani *MIT. This gene was originally annotated as 'sugar transporter, putative' in both the original GenBank annotation file and in the GeneDB entry for LmjF24.0680. We manually annotated LmjF24.0680 as the predicted *L. major *MIT, and assigned the evidence code indicating that the inference was computational. In addition, for every gene in LeishCyc, we have added a link to the corresponding entry in GeneDB which directly connects the entries from the two databases.

### Curation of protein-linked reactions

Literature searches have identified a number of genes that have been linked to a particular enzyme or transporter in *Leishmania *spp. or other trypanosomatids. Such genes were identified in LeishCyc, checked as to whether they were linked to the correct reaction(s), and, if not, the respective entries were corrected.

Some enzymes were associated with an incorrect EC number in the *L. major *Genome Project annotation file, resulting in the enzyme being linked to incorrect reaction(s). For example, the phosphomannomutase enzyme catalyzes the reaction EC 5.4.2.8 (α-D-mannose 1-phosphate → mannose 6-phosphate), but in the annotation file it was associated with EC 3.1.3.11 and thus was linked to the EC 3.1.3.11 reaction (fructose 1,6-bisphosphate + H_2_O → fructose 6-phosphate + P_i_). In the subsequent manual curation, phosphomannomutase was removed from the EC 3.1.3.11 reaction and linked to the EC 5.4.2.8 reaction. In total, we found 7 enzymes to be annotated with the incorrect EC number in the *L. major *genome file, and we linked these enzymes to the correct reactions.

For genes not associated with an EC number in the *L. major *genome annotation file, the Pathway Tools software has attempted to link the gene based on matches of the product name to enzyme function. For example, the gene LmjF15.1010 was annotated as glutamate dehydrogenase and was matched to three glutamate dehydrogenase reactions each with a different EC number (EC 1.4.1.2, EC 1.4.1.3 and EC 1.4.1.4). The three reactions only differ in the cofactor used:

EC 1.4.1.2: L-glutamate + NAD^+ ^+ H_2_O → α-ketoglutarate + ammonia + NADH

EC 1.4.1.3: L-glutamate + NAD(P)^+ ^+ H_2_O → α-ketoglutarate + ammonia + NAD(P)H

EC 1.4.1.4: L-glutamate + NADP^+ ^+ H_2_O → α-ketoglutarate + ammonia + NADPH

LmjF15.1010 is the predicted ortholog of the *L. tarentolae *mitochondrial glutamate dehydrogenase [33]. The *L. tarentolae *enzyme uses NAD^+ ^as a cofactor, but also has an NADP^+ ^binding site, and so, in this case, the linkage of LmjF15.1010 to EC 1.4.1.3 was kept, and removed from EC 1.4.1.2 and E.C. 1.4.1.4.

In certain cases, manual intervention was required to link enzymes to the multiple reactions they catalyze. For example, the enzyme pteridine reductase was associated with EC 1.5.1.33 and was thus automatically linked to only one reaction. However, this enzyme has been shown to catalyze additional reactions in folate and biopterin metabolism in *L. major *[34], and we manually linked pteridine reductase to these additional (three) reactions. Similarly, the enzyme trypanothione synthetase was associated with EC 6.3.1.9 and automatically linked to one reaction. However, it has been demonstrated experimentally that this enzyme also catalyzes EC 6.3.1.8 [35], thus we linked trypanothione synthetase to this reaction as well.

For newly discovered enzymes without annotation in the original genome project files, the required enzyme objects were manually linked with the relevant reactions and, if necessary, the reaction in question was created. For example, the *L. major *inositol phosphorylceramide (IPC) synthase gene was recently identified by [36]. This gene was listed as a hypothetical protein in the *L. major *genome annotation file. We changed its annotation in LeishCyc to 'inositol phosphorylceramide synthase', manually created a new reaction (ceramide + L-1-phosphatidylinositol → inositol phosphorylceramide + 1,2-diacylglycerol), and linked the product of the gene to this reaction.

In addition to metabolic enzymes, a concerted identification and curation of transport reactions was performed. After the automated build, there were 23 transporters identified in LeishCyc, but only 6 transport reactions. Furthermore, many of the identified transporters had not been assigned specific transporter identities (e.g. MIT). Using the literature, we identified a further 26 *L. major *transporters (making 49 in total) and created 31 transport reactions [see Additional file 3].

### Assignment of evidence codes and citations

**Figure 1:**
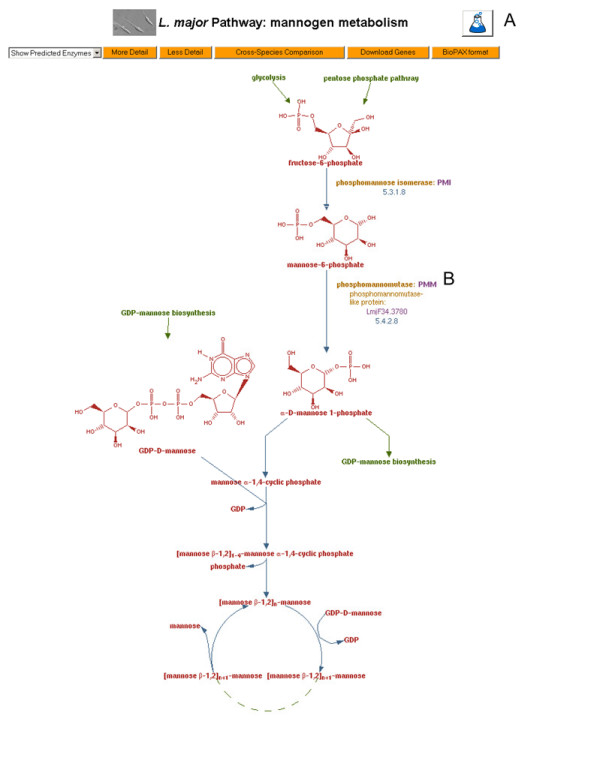
**Example of a LeishCyc pathway with evidence codes**. This pathway for mannogen (formerly termed 'mannan') metabolism is a new pathway experimentally identified in *L. major *[31,42]. A: the flask icon indicates that there is experimental evidence for this pathway in *Leishmania*; B: Enzymes with names in bold type signify that there is experimental evidence that the *Leishmania *protein catalyzes the reaction shown. Enzymes in regular type have either computational evidence alone or no associated evidence at all. Arrows and dashed lines represent reactions and polymerisation reactions, respectively.

The BioCyc ontology allows evidence codes to be assigned to support assertions in the BioCyc type database [37]. If the supporting evidence was experimental, an evidence icon of a flask appears in the Pathway Tools software visual representation, with the assigned evidence code being 'EV-EXP' (see Figure 1). In LeishCyc this evidence code was manually assigned to signify experimental evidence, when the evidence came from any of the *Leishmania *spp. If the supporting evidence was only computational the evidence code 'EV-COMP' was assigned (this type of evidence is shown as a computer icon). If the evidence is supported by a publication, alongside each evidence code there is a link to the supporting publication. The code for curated proteins shows the evidence that supports the association of the protein with its linked reaction (i.e. for enzymes, this is the evidence that the enzyme catalyzes a given reaction or, for transporters, that the protein transports a particular substrate). In cases where the *L. major *ortholog was identified by the LeishCyc curator from similarity to a published sequence, the evidence code assigned was 'EV-COMP-HINF-FN-FROM-SEQ' (human inference of function from sequence), with additional explanations and the percent identity that the *L. major *sequence shares with the published sequence given. Currently, 208 proteins (including 2 protein complexes), 254 reactions, and 130 pathways have been assigned evidence codes in LeishCyc, and 200 references have been added to the database (Table 1). An example of a LeishCyc metabolic pathway with evidence icons and codes as displayed by the Pathway Tools software is shown in Figure 1.

## Utility

LeishCyc provides a platform for curation, refinement, and dissemination of information about *Leishmania *metabolic pathways incorporating extensive manual curation and supported by the extensive query and visualization functionality of the Pathway Tools software [25]. The underlying BioCyc ontology provides a detailed, well-developed ontology designed to capture biological function [14,38], and to maximize accuracy of resulting repositories [13]. Furthermore, the same ontology is used in a number of other organism-specific databases [16,19-24], which opens the possibility for accurate cross-organism comparisons based on the biochemical components and associated abstract entities, such as reactions and metabolic pathways. LeishCyc enables the overlay of experimental data from genome-wide studies onto the visual representations of the *L. major *biochemical network. This can be achieved by the Omics Viewer component of the Pathway Tools software, which provides the ability to visualize high-throughput ('omics') data sets within the LeishCyc cellular overview diagram [39]. Three examples of LeishCyc utility in data visualization and analysis are described below.

### Visualization of proteomics data

**Figure 2:**
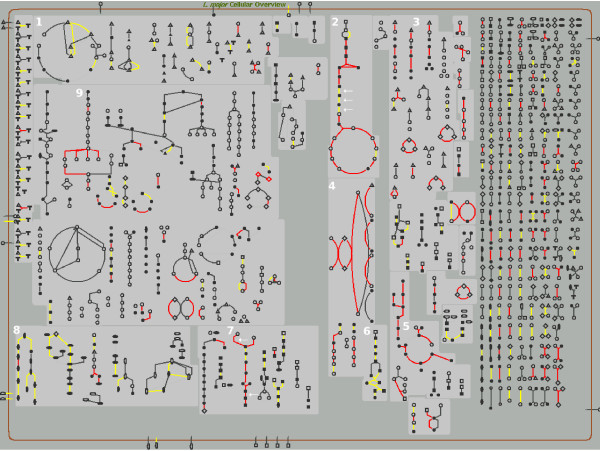
**Overlay of proteomic data sets using the LeishCyc Omics Viewer**. Changes in the proteome of *L. donovani *promastigotes and axenic amastigotes [40] were mapped on to the Cellular Overview using the Pathway Tools Omics Viewer functionality. The supplied gene IDs for *L. donovani*/*L. infantum *were converted to the corresponding *L. major *orthologs prior to mapping. Lines (representing proteins) coded in red represent enzymes that were increased in amastigotes, lines in yellow represent enzymes that were decreased. Selected pathways/pathway groups are numbered as follows: 1 = amino acid biosynthesis; 2 = glycolysis and the tricarboxylic acid (TCA) cycle; 3 = amino acid catabolism; 4 = oxidative phosphorylation; 5 = fatty acid beta oxidation; 6 = pentose phosphate pathway; 7 = gluconeogenesis; 8 = nucleotide biosynthesis; 9 = ergosterol biosynthesis. The arrows in the glycolysis and gluconeogenesis pathways are enzymes specific to these pathways [40].

Figure 2 shows alterations in protein expression levels during the differentiation of *L. donovani *promastigotes to *in vitro *differentiated (axenic) amastigotes [40], mapped onto the LeishCyc pathways. Enzymes that are decreased in the amastigote stage are shown in yellow while those that are increased are shown in red. Decreases in expression levels of enzymes involved in glycolysis (3 enzymes) and the pentose phosphate pathway (6 enzymes) are apparent, as are increases in enzymes involved in gluconeogenesis (2 enzymes), oxidative phosphorylation (4 enzymes), amino acid catabolism (3 enzymes), and fatty acid β-oxidation (5 enzymes). Additional patterns in the data become apparent in this representation, including decreases in some enzymes involved in nucleotide biosynthesis (8 enzymes), and increases in enzymes of ergosterol biosynthesis (9 enzymes). The use of LeishCyc greatly reduced the time needed to produce representations of these stage-specific metabolic changes. Furthermore, the Omics Viewer can be used to display the time points as a progressive series of images, creating an effect of data animation [see Additional file 4].

### Visualization of metabolomics data

**Figure 3:**
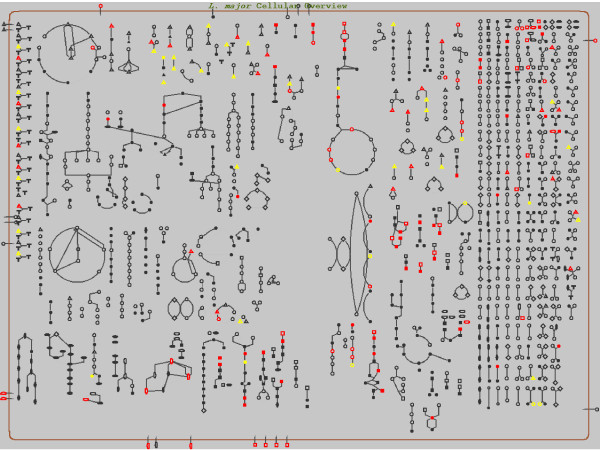
**Overlay of metabolomic data sets using the LeishCyc Omics Viewer**. Polar metabolites in *L. mexicana *promastigotes and axenic amastigotes were detected by GC-MS (see the main text for details). Metabolites (represented by shapes) in red increase in amastigote stages, while those that decrease are in yellow.

In the second example, LeishCyc was used to visualize data from metabolic profiling experiments performed in our group (Figure 3). *L. mexicana *MZ 379 promastigotes were cultured in RPMI 1640 medium supplemented with 10% heat-inactivated foetal bovine serum (iFBS) at 27°C. Log phase promastigotes were harvested approximately two days after inoculation of the media. Axenic amastigotes were generated from stationary phase parasites (5–6 days after passage) by adjusting the conditioned media to pH 5.5 with HCl and the addition of iFBS to 20% [41,42]. The adjusted culture was incubated at 33°C and amastigote-like forms of the parasite were harvested on days 5 and 6. Parasite metabolism was quenched and polar metabolites extracted, derivatized, and analyzed by gas chromatography-mass spectrometry (GC-MS), as described previously [43]. Figure 3 shows a colour-coded representation of changes in metabolite steady-state concentrations in axenic amastigotes relative to log-phase promastigotes. Significant changes in the steady state levels of many metabolites were detected. In particular, the levels of hexose phosphates and intermediates in glycolysis were reduced, while levels of many amino acids increased. Such a representation of metabolomic data in the context of the cellular biochemical pathways is highly useful for the observation of patterns in relative changes. In addition, the built-in capabilities of Pathway Tools allow one to interactively interrogate the organism metabolic pathways map overlaid with experimental data (such as those shown in Figures 2 and 3), in order to investigate observed patterns.

### Network chokepoint analysis

Chokepoint analysis has been used to prioritize potential drug targets in the *P. falciparum *PlasmoCyc database [22]. Chokepoints are defined as reactions that consume unique substrates or produce unique products, potentially important criteria for drug target prioritization [22,44]. Pathway Tools was used to identify 324 chokepoint reactions in LeishCyc. These reactions have 145 enzymes associated with them, corresponding to 132 genes [see Additional file 5]. This list includes a number of enzymes previously predicted to be essential for normal growth or infectivity. Included in the list is lanosterol 14α-demethylase (LmjF11.1100), the protein target of ketoconazole, the only clinical drug for leishmaniasis with an identified protein target [45]. The TDR Targets database [46] was used to identify 31 genes in this list that do not have human orthologs [see Additional file 5]. Interestingly, a number of the genes identified in the chokepoint analysis of LeishCyc were also identified in a corresponding analysis of *P. falciparum *[22].

## Discussion and conclusion

LeishCyc captures the information about *Leishmania *metabolic pathways from genome annotations and literature sources, and organizes this information into a structured database supported by a well developed, publicly available ontology [13-15,47]. LeishCyc provides a systematic approach to organizing the evolving knowledge about *Leishmania *biochemical networks, as well as tools for analysis, interpretation, and visualization of *Leishmania *high-throughput ('omics') data in the context of metabolic pathways. We believe that LeishCyc provides an important new resource for analysis and construction of metabolic network models for these parasites, for mapping species- or stage-specific changes in transcript, protein and metabolite levels, and for prioritizing potential drug candidates by metabolic network analysis. LeishCyc advanced search features and Omics Viewer capabilities can be accessed through a standard web browser, and the LeishCyc content is provided based on the Creative Commons license .

It is believed that 24 species of *Leishmania *infect humans. The genome of *L. major *was the first to be completely sequenced, and was used as the basis for the initial build of LeishCyc [4]. While different species of *Leishmania *can exhibit distinct trophism for different sandfly species, and induce a spectrum of disease in humans and other mammalian hosts, recent sequencing projects have highlighted a remarkable degree of synteny and conservation across the genomes of three major pathogenic species [6,48]. Only a very limited number of genes were shown to be present in one, but not other species of *Leishmania*. The metabolic networks identified in LeishCyc are therefore likely to be relevant to all species of *Leishmania*. Interestingly, recent studies have suggested that species specific differences in gene transcription or protein expression may underlie some of the differences in biology and disease phenotypes of different *Leishmania *species [48-50]. As demonstrated in this study, the LeishCyc tools can be used to visualize global changes in protein expression patterns in different developmental stages, and this type of analysis can be readily extended to identify differences in transcript and protein expression levels in different species.

Another important feature of LeishCyc is the capacity to overlay metabolite profiling data sets on the predicted metabolic networks. *Leishmania *and other trypanosomatids are unusual in lacking a conventional network of transcriptional factors and most protein encoding genes are constitutively transcribed in all life cycle stages [51]. Consequently, *Leishmania *metabolism may be largely regulated by post-translational mechanisms. In particular, it is likely that changes in external nutrients and scavenging pathways, and allosteric regulation of intracellular enzymes may play key roles in regulating metabolic processes [3]. As shown in this study, differences in the metabolite profiles of promastigote (insect stage) and axenic amastigotes (mammalian-infective stages) of *L. mexicana *can be mapped onto the LeishCyc metabolic network, providing an important tool for both identifying stage-specific changes in metabolism and assessing the extent to which these changes correlate with transcript or protein levels.

The LeishCyc database organizes the existing knowledge about *Leishmania *biochemical reactions, gene products and metabolites into metabolic pathways in a mathematically well defined manner that will serve as the foundation for *Leishmania *systems studies, including computer-aided reconstructions of metabolic networks. Such a reconstruction of the *L. major *metabolic network has recently been reported by Papin and colleagues [7]. The *L. major *iAC560 metabolic network reconstruction included 560 genes (6.7% of the genome) and an additional 103 predicted gene associated reactions that were added for proper functioning of the computational model. The latter remain to be experimentally verified. Interestingly, the iAC560 metabolic network reconstruction was only partially successful in predicting a number of experimentally observed properties of *Leishmania *metabolism, such as minimal amino acid requirements and the potential lethality of single gene deletions [7], highlighting significant gaps in this model. In this respect, it is notable that the curated LeishCyc database contains more than 1074 genes that encode enzymatic or transport reactions, even after removal of most incomplete pathways. LeishCyc is therefore likely to constitute an important resource for refining metabolic reconstructions in the future. A similar database for a related organism, *T. brucei*, is currently under development [52]. A collection of such databases will provide an unprecedented platform for detailed comparative studies of organisms from the Trypanosomatidae family that can be accessed and queried programmatically through Application Programming Interfaces (APIs) exposed by Pathway Tools [53].

## Availability and requirements

LeishCyc is available on the Internet from URL: 

## Abbreviations

KEGG: Kyoto Encyclopedia of Genes and Genomes; GPI: glycosylphosphatidylinositol; MIT: *myo*-inositol transporter; iFBS: heat-inactivated foetal bovine serum; GC-MS: gas chromatography-mass spectrometry; TCA: tricarboxylic acid.

## Authors' contributions

The LeishCyc project was established by VAL and MJM. MAD performed the main curation work of LeishCyc during the initial development, including manual editing of the database, literature searches, and bioinformatics studies with VAL. JIM was involved in the database curation. DPDS and ECS were involved in metabolite profiling experiments of *Leishmania *parasites. MJM was involved in guidance in the development of LeishCyc and, in particular, was the chief *Leishmania *biology adviser. VAL was responsible for all bioinformatics aspects of the project. MAD and VAL have drafted the initial manuscript. MJM, MAD, JIM, VAL have been involved in revising the manuscript. All authors have read and approved the final manuscript.

## Supplementary Material

Additional file 1 **LeishCyc pathways created or modified after the initial build**. Contains a table summarizing LeishCyc pathways created or modified after the initial build. Click here for file

Additional file 2 **Pathways removed from LeishCyc after the initial build**. Contains a table showing pathways were manually removed because either there was weak evidence for their existence in *Leishmania *spp. or they were replaced with a *Leishmania*-specific pathway. Click here for file

Additional file 3 **Transporters in Leishmania**. Transporters and transport reactions identified in LeishCyc. Shown are the enzymes linked to those reactions. Details of transporter synonyms and localization (predicted and experimentally verified) are also indicated. Click here for file

Additional file 4 **Time course proteomic experiment shown in LeishCyc OmicsViewer**. Colour-coded representation of changes in steady-state protein levels during promastigote differentiation into amastigotes. The data is from the time course experiments of Rosenzweig et al. (Rosenzweig D, Smith D, Opperdoes F, Stern S, Olafson RW, Zilberstein D, "Retooling *Leishmania *metabolism: from sand fly gut to human macrophage", Faseb J 2008, 22(2):590–602), with 2.5 h, 5 h, 10 h, 15 h, 24 h (promastigote), and 144 h (amastigote) time points shown. Changes are shown relative to the 0 h time point (promastigote). Metabolites and proteins are represented by shapes and lines, respectively. Proteins that are decreased are shown in yellow, those increased in red, and those unchanged in blue. Click here for file

Additional file 5 **LeishCyc chokepoint reactions**. Chokepoint reactions were identified for *L. major *using the Pathway Tools chokepoint identification tool. Shown are the enzymes linked to those reactions. Enzymes are grouped within major pathway categories and those with human orthologs are indicated (as determined by the TDR Targets database, with ortholog groupings from OrthoMCL v1.2). Click here for file
