# Supplementary material for: LeishCyc: a biochemical pathways database for Leishmania major
Source: BMC Syst Biol. 2009 Jun 5;3:57. doi: 10.1186/1752-0509-3-57 (PMC2700086; doi:10.1186/1752-0509-3-57)
Supplement: Additional file 1 — LeishCyc pathways created or modified after the initial build. Contains a table summarizing LeishCyc pathways created or modified after the initial build. [file 1752-0509-3-57-S1.doc]

"LeishCyc: a biochemical pathways database for *Leishmania major*" Maria A. Doyle, James I. MacRae, David P. De Souza, Eleanor C. Saunders, Malcolm J. McConville, and Vladimir A. Likic

| 2-oxobutanoate degradation I |
| --- |
| aerobic respiration - electron donor |
| acetyl-CoA degradation to acetate |
| ascorbate biosynthesis |
| asparagine biosynthesis |
| asparagine degradation |
| aspartate degradation |
| biopterin metabolism |
| citrate degradation |
| dolichol biosynthesis |
| dolichyl-diphosphooligosaccharide biosynthesis |
| ergosterol biosynthesis |
| ester phospholipid biosynthesis |
| ether phospholipid biosynthesis |
| fatty acid biosynthesis - elongase pathway |
| fatty acid elongation - unsaturated |
| folate metabolism |
| GDP-arabinose biosynthesis |
| GDP-mannose biosynthesis |
| gluconeogenesis |
| glutamate degradation |
| glutathionylspermidine biosynthesis |
| glycerol metabolism |
| glycerolipid biosynthesis - initial steps |
| glycoinositolphospholipid (GIPL) biosynthesis |
| glycolysis |
| GPI anchor biosynthesis |
| glyoxalase pathway |
| heme biosynthesis |
| L-serine degradation |
| leucine degradation I |
| mannogen metabolism |
| methionine biosynthesis |
| methionine salvage pathway |
| nitric oxide biosynthesis |
| ornithine biosynthesis (arginine degradation) |
| ovothiol A biosynthesis |
| oxaloacetate degradation to pyruvate |
| phenylalanine degradation |
| polyisoprenoid biosynthesis |
| polyphosphate metabolism |
| proline degradation |
| purine nucleotide metabolism (phosphotransfer and nucleotide modification) |
| pyrimidine nucleotide metabolism (phosphotransfer and nucleotide modification) |
| S-adenosyl-L-methionine biosynthesis |
| S-adenosyl-L-methionine cycle |
| salvage pathways of purine nucleosides |
| salvage pathways of pyrimidine ribonucleotides |
| succinate fermentation pathway |
| superpathway of glycolysis, pyruvate dehydrogenase and the TCA cycle |
| superpathway of mitochondrial fatty acid biosynthesis |
| superpathway of phospholipid biosynthesis |
| superpathway of polyamine biosynthesis I |
| superpathway of S-adenosylmethionine metabolism |
| superpathway of sterol biosynthesis |
| superpathway of threonine degradation |
| threonine degradation I |
| threonine degradation II |
| triacylglycerol biosynthesis |
| trypanothione redox reactions |
| tryptophan degradation |
| ubiquinone-9 biosynthesis |
| UDP-galactose biosynthesis |
| UDP-*N*-acetylglucosamine biosynthesis |
