# Supplementary material for: LeishCyc: a biochemical pathways database for Leishmania major
Source: BMC Syst Biol. 2009 Jun 5;3:57. doi: 10.1186/1752-0509-3-57 (PMC2700086; doi:10.1186/1752-0509-3-57)
Supplement: Additional file 2 — Pathways removed from LeishCyc after the initial build. Contains a table showing pathways were manually removed because either there was weak evidence for their existence in Leishmania spp. or they were replaced with a Leishmania-specific pathway. [file 1752-0509-3-57-S2.doc]

"LeishCyc: a biochemical pathways database for *Leishmania major*" Maria A. Doyle, James I. MacRae, David P. De Souza, Eleanor C. Saunders, Malcolm J. McConville, and Vladimir A. Likic

| acetyl-CoA fermentation to butyrate |
| --- |
| aerobic respiration - electron donor II |
| β-alanine biosynthesis I |
| β-alanine biosynthesis II |
| allantoin degradation I |
| arginine biosynthesis III |
| arginine biosynthesis IV |
| arginine degradation I (arginase pathway) |
| arginine degradation II |
| arginine degradation III (arginine decarboxylase/agmatinase pathway) |
| arginine degradation VI (arginase 2 pathway) |
| ascorbate glutathione cycle |
| asparagine biosynthesis III |
| asparagine degradation I |
| asparagine degradation II |
| aspartate degradation II |
| bitter acids biosynthesis |
| brassinosteroid biosynthesis II |
| C4 photosynthetic carbon assimilation cycle |
| canavanine biosynthesis |
| canavanine degradation |
| cholesterol biosynthesis I |
| cholesterol biosynthesis II (via 24,25-dihydrolanosterol) |
| cholesterol biosynthesis III (via desmosterol) |
| citrulline biosynthesis |
| citrulline-nitric oxide cycle |
| cohumulone biosynthesis |
| colanic acid building blocks biosynthesis |
| cyanate degradation |
| D-camphor degradation |
| D-galactarate degradation |
| D-glucarate degradation |
| D-lactate fermentation to propionate and acetate |
| dopamine degradation |
| dTDP-L-rhamnose biosynthesis I |
| dTDP-L-rhamnose biosynthesis II |
| enterobacterial common antigen biosynthesis |
| Entner-Doudoroff pathway II (non-phosphorylative) |
| Entner-Doudoroff pathway III (semi-phosphorylative) |
| epicuticular wax biosynthesis |
| ethylene glycol degradation |
| fatty acid elongation -- unsaturated I |
| fatty acid α-oxidation |
| fatty acid β-oxidation |
| flavonoid biosynthesis |
| folate polyglutamylation II |
| folate transformations |
| formaldehyde assimilation I (serine pathway) |
| formaldehyde assimilation II (RuMP Cycle) |
| formaldehyde oxidation I |
| formyl-tetrahydrofuran (-THF) biosynthesis II |
| fucose degradation |
| galactose degradation II |
| gallate degradation III (anaerobic) |
| geranyldiphosphate biosynthesis |
| glucose and glucose 1-phosphate degradation |
| glucose fermentation to lactate II |
| glucose heterofermentation to lactate I |
| glutamate biosynthesis III |
| glutaryl-CoA degradation |
| glycerol degradation I |
| glycerol degradation IV |
| glycine betaine biosynthesis II (Gram-positive bacteria) |
| glycogen degradation |
| glycolate and glyoxylate degradation I |
| glycolysis I |
| glycolysis III |
| heme biosynthesis II |
| humulone biosynthesis |
| isoleucine degradation II |
| L-cysteine degradation I |
| L-cysteine degradation III |
| lactate biosynthesis |
| leucine degradation III |
| linear furanocoumarin biosynthesis |
| methionine biosynthesis I |
| methionine biosynthesis II |
| methionine degradation III |
| methylglyoxal degradation I |
| methylglyoxal degradation IV |
| methylglyoxal degradation V |
| mixed acid fermentation |
| naphthalene degradation |
| O-antigen biosynthesis |
| octane oxidation |
| pentose phosphate pathway (partial) |
| phenylalanine degradation I |
| phenylalanine degradation III |
| phenylpropanoid biosynthesis |
| phospholipid biosynthesis I |
| phospholipid biosynthesis II |
| proline biosynthesis I |
| proline degradation II |
| purine degradation |
| purine fermentation to acetate and CO_2_ |
| purine nucleotides *de novo* biosynthesis I |
| putrescine biosynthesis I |
| putrescine degradation III |
| pyruvate fermentation to propionate |
| reductive acetyl-coA pathway |
| rhamnose degradation |
| rosmarinic acid biosynthesis I |
| S-adenosylmethionine biosynthesis |
| salvage pathways of adenine, hypoxanthine, and their nucleosides |
| salvage pathways of guanine, xanthine, and their nucleosides |
| salvage pathways of purine and pyrimidine nucleotides |
| salvage pathways of purine nucleosides II (plant) |
| starch biosynthesis |
| starch degradation |
| sterol biosynthesis |
| suberin biosynthesis |
| sucrose degradation to ethanol and lactate (anaerobic) |
| superpathway of citrulline metabolism |
| superpathway of D-glucarate and D-galactarate degradation |
| superpathway of fucose and rhamnose degradation |
| superpathway of saturated and unsaturated fatty acid elongation |
| TCA cycle variation II |
| tetrahydrofolate biosynthesis II |
| trehalose degradation V |
| ubiquinone biosynthesis I (aerobic) |
| UDP-galactose biosynthesis (salvage pathway from galactose using UDP-glucose) |
| UDP-glucose conversion |
| UDP-*N*-acetylgalactosamine biosynthesis |
| valine degradation II |
| xanthohumol biosynthesis |
