# Supplementary material for: LeishCyc: a biochemical pathways database for Leishmania major
Source: BMC Syst Biol. 2009 Jun 5;3:57. doi: 10.1186/1752-0509-3-57 (PMC2700086; doi:10.1186/1752-0509-3-57)

This document contains Supplementary Figure 1 for the manuscript "LeishCyc: a biochemical pathways database for *Leishmania major*" Maria A. Doyle, James I. MacRae, David P. De Souza, Eleanor C. Saunders, Malcolm J. McConville, and Vladimir A. Likic

Supplementary Figure 1

Colour-coded representation of changes in steady-state protein levels during promastigote differentiation into amastigotes. This data is from the time course experiments of Rosenzweig et al. (Rosenzweig D, Smith D, Opperdoes F, Stern S, Olafson RW, Zilberstein D: Retooling Leishmania metabolism: from sand fly gut to human macrophage. Faseb J 2008, 22(2):590-602), with 2.5 h, 5 h, 10 h, 15 h, 24 h (all promastigote), and 144 h (amastigote) time points shown. Changes are shown relative to the 0 h time point (promastigote). Metabolites and proteins are represented by shapes and lines, respectively. Proteins that are decreased are shown in yellow, those increased in red, and those unchanged in blue.


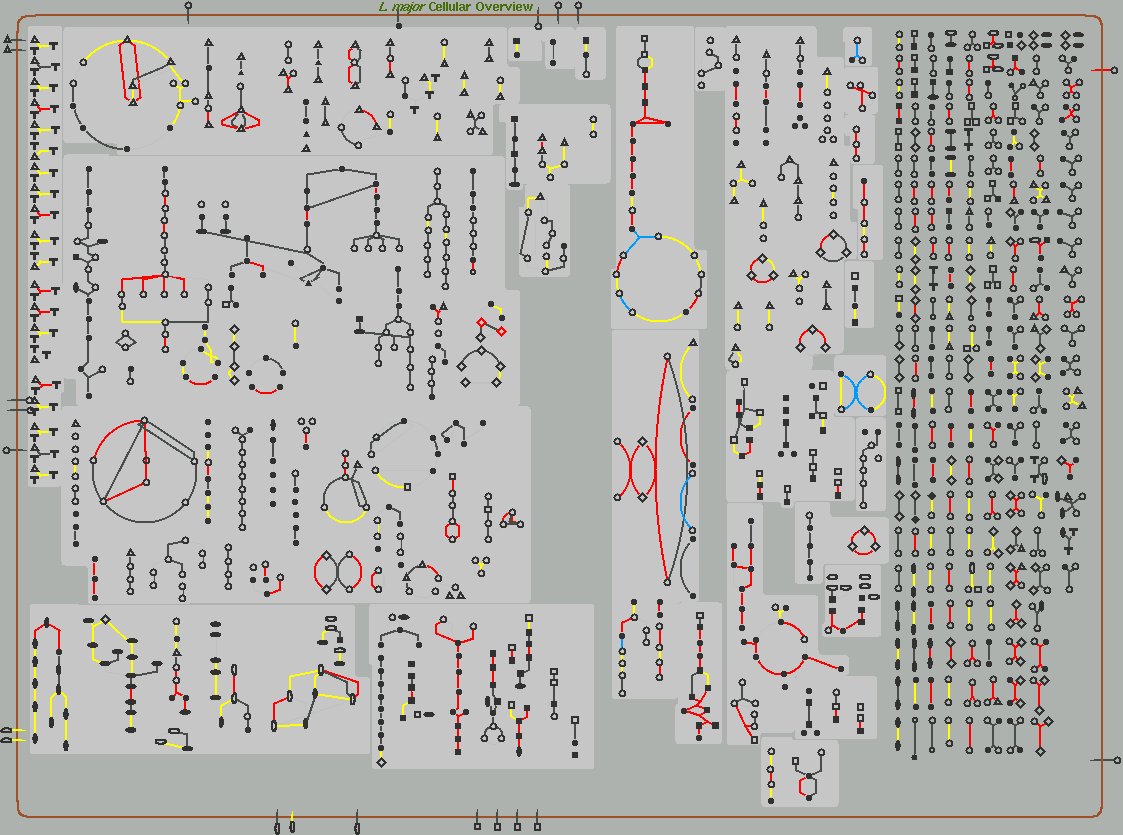
Time point: 2.5 h (promastigote)

Time point: 5 h
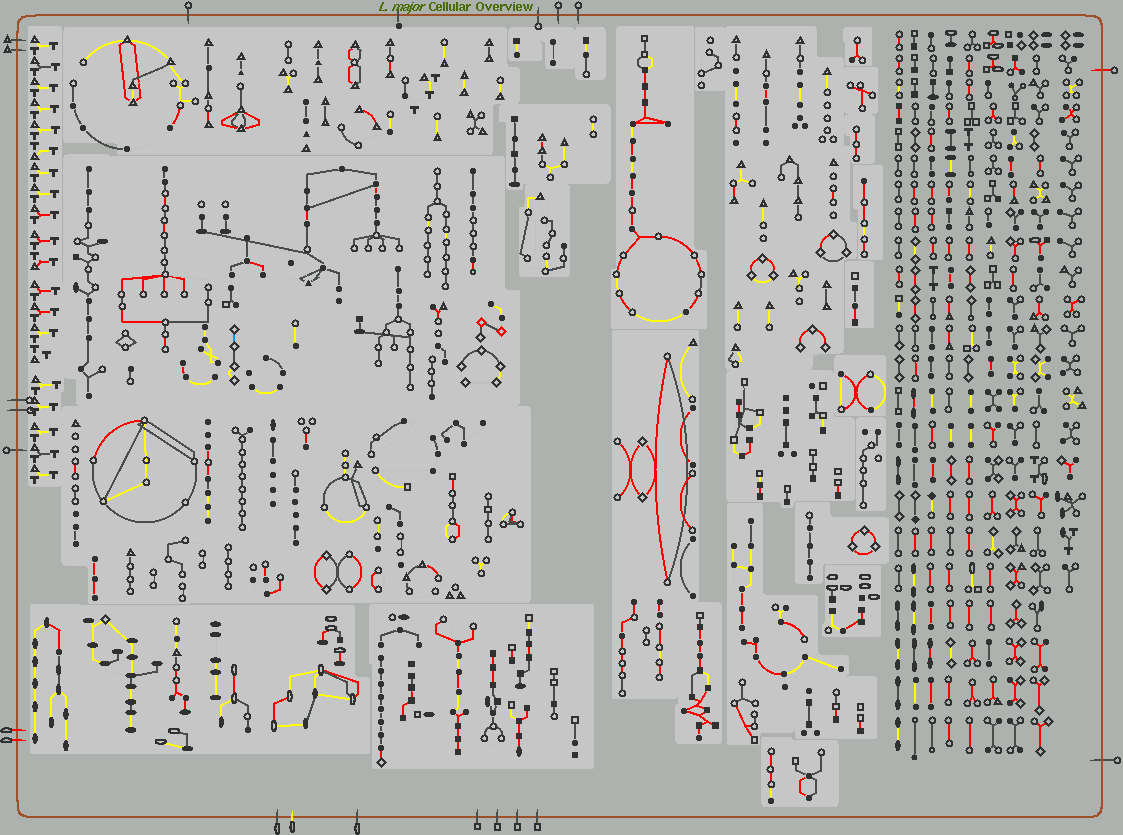
(promastigote)

Time point: 10 h (promastigote)


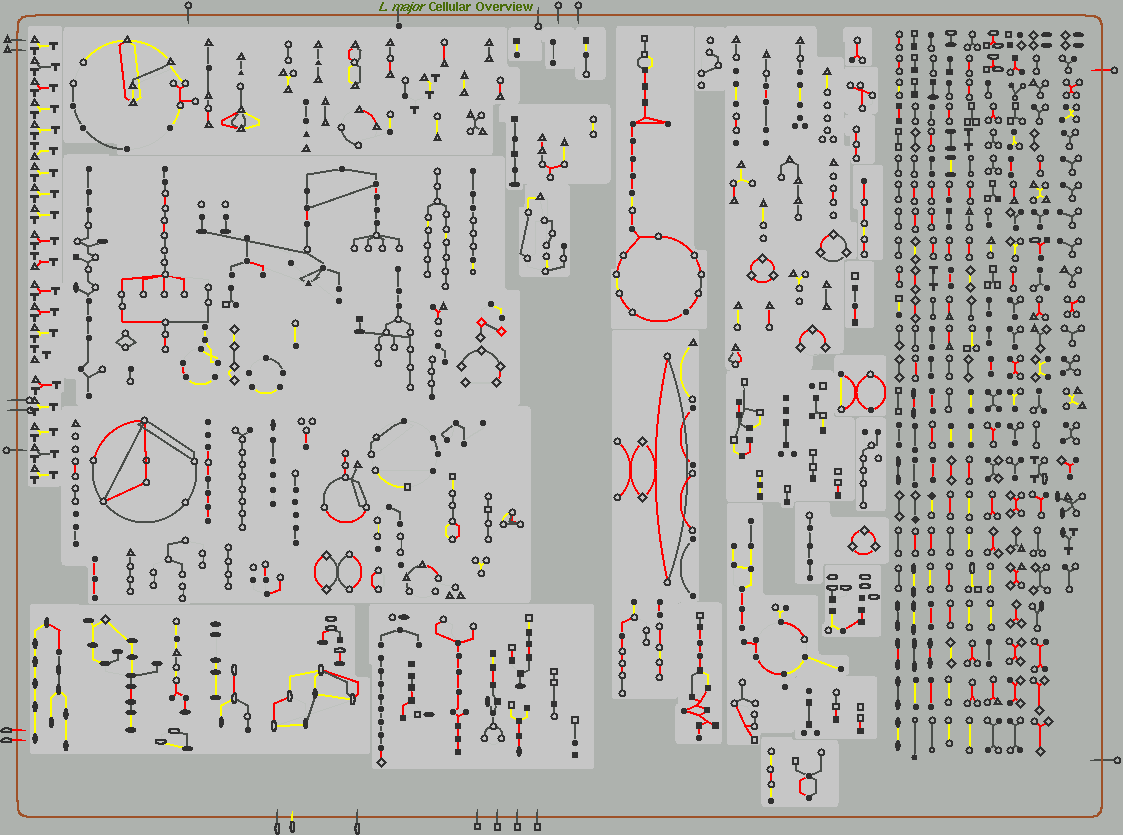


Time point: 15 h (promastigote)


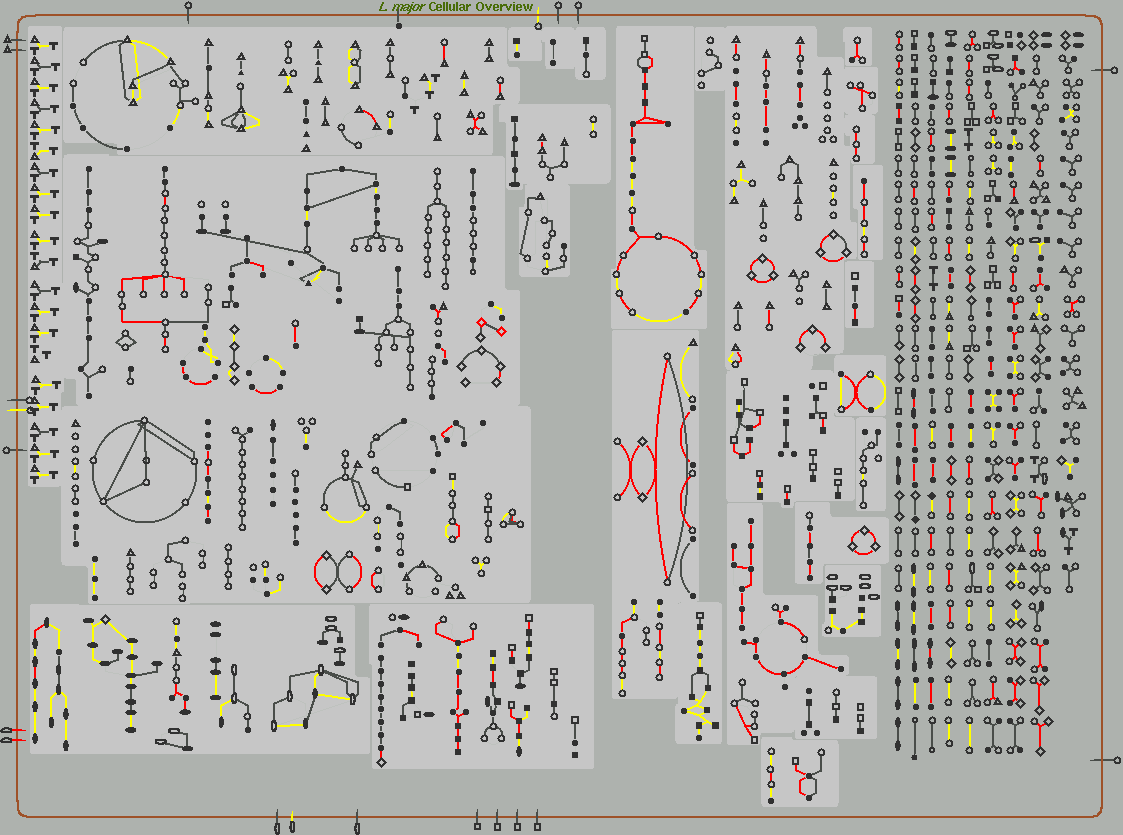


Time point: 24 h (promastigote)


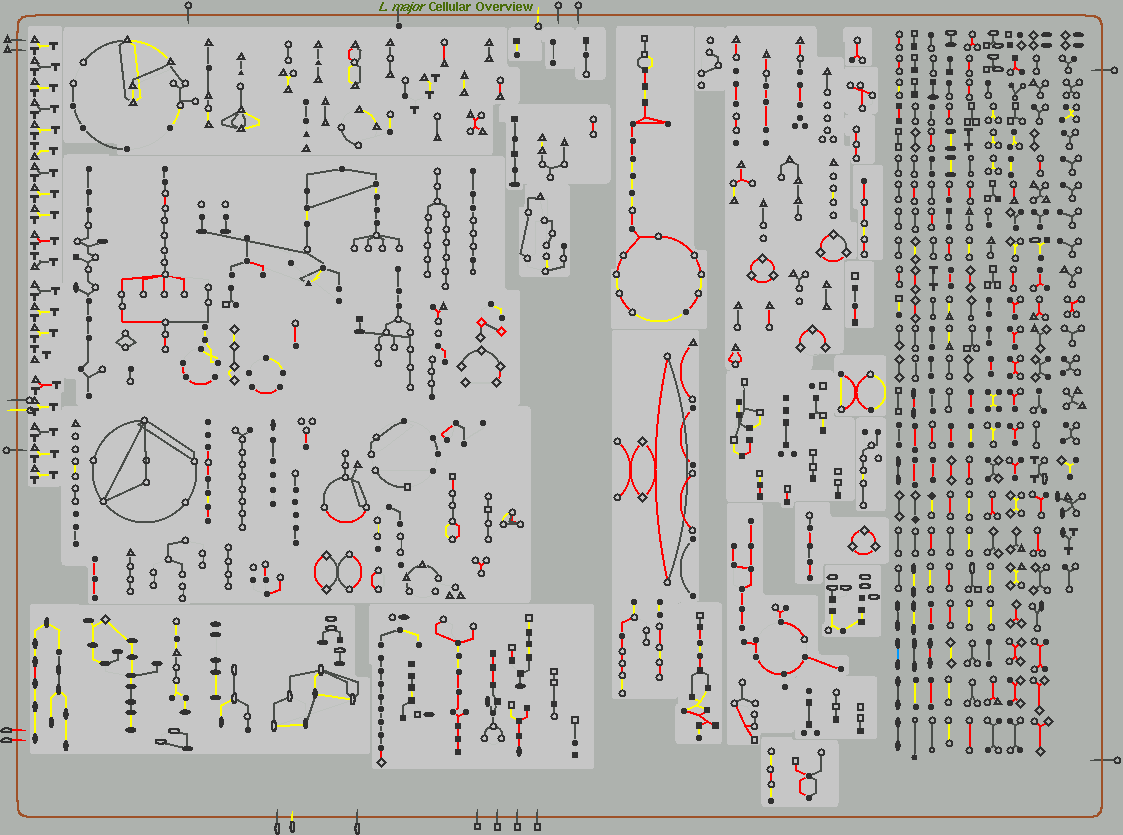


Time point: 144 h (amastigote)


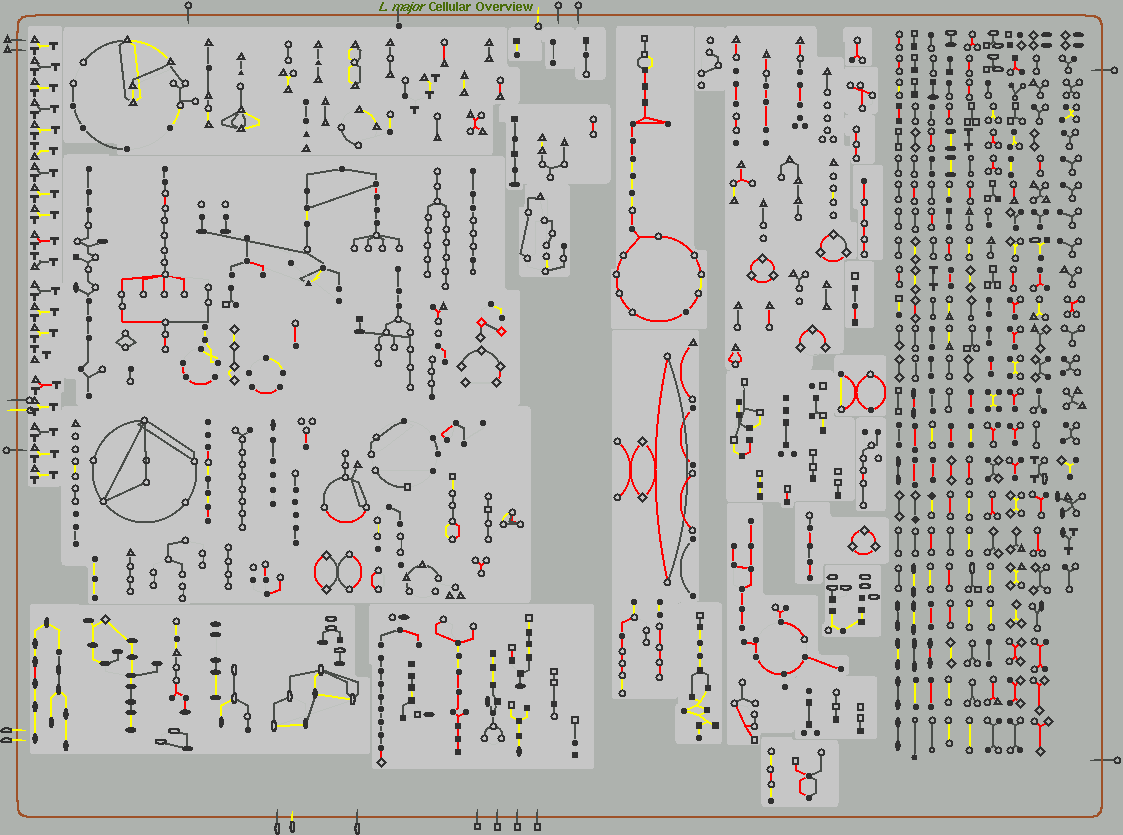

Supplement: Additional file 4 — Time course proteomic experiment shown in LeishCyc OmicsViewer. Colour-coded representation of changes in steady-state protein levels during promastigote differentiation into amastigotes. The data is from the time course experiments of Rosenzweig et al. (Rosenzweig D, Smith D, Opperdoes F, Stern S, Olafson RW, Zilberstein D, "Retooling Leishmania metabolism: from sand fly gut to human macrophage", Faseb J 2008, 22(2):590–602), with 2.5 h, 5 h, 10 h, 15 h, 24 h (promastigote), and 144 h (amastigote) time points shown. Changes are shown relative to the 0 h time point (promastigote). Metabolites and proteins are represented by shapes and lines, respectively. Proteins that are decreased are shown in yellow, those increased in red, and those unchanged in blue. [file 1752-0509-3-57-S4.doc]
